# Building a National Neighborhood Dataset From Geotagged Twitter Data for Indicators of Happiness, Diet, and Physical Activity

**DOI:** 10.2196/publichealth.5869

**Published:** 2016-10-17

**Authors:** Quynh C Nguyen, Dapeng Li, Hsien-Wen Meng, Suraj Kath, Elaine Nsoesie, Feifei Li, Ming Wen

**Affiliations:** ^1^ Department of Health, Kinesiology, and Recreation University of Utah College of Health Salt Lake City, UT United States; ^2^ Department of Geography University of Utah Salt Lake City, UT United States; ^3^ School of Computing University of Utah Salt Lake City, UT United States; ^4^ Department of Global Health University of Washington Seattle, WA United States; ^5^ Department of Sociology University of Utah Salt Lake City, UT United States

**Keywords:** social media, Twitter messaging, health behavior, happiness, food, physical activity

## Abstract

**Background:**

Studies suggest that where people live, play, and work can influence health and well-being. However, the dearth of neighborhood data, especially data that is timely and consistent across geographies, hinders understanding of the effects of neighborhoods on health. Social media data represents a possible new data resource for neighborhood research.

**Objective:**

The aim of this study was to build, from geotagged Twitter data, a national neighborhood database with area-level indicators of well-being and health behaviors.

**Methods:**

We utilized Twitter’s streaming application programming interface to continuously collect a random 1% subset of publicly available geolocated tweets for 1 year (April 2015 to March 2016). We collected 80 million geotagged tweets from 603,363 unique Twitter users across the contiguous United States. We validated our machine learning algorithms for constructing indicators of happiness, food, and physical activity by comparing predicted values to those generated by human labelers. Geotagged tweets were spatially mapped to the 2010 census tract and zip code areas they fall within, which enabled further assessment of the associations between Twitter-derived neighborhood variables and neighborhood demographic, economic, business, and health characteristics.

**Results:**

Machine labeled and manually labeled tweets had a high level of accuracy: 78% for happiness, 83% for food, and 85% for physical activity for dichotomized labels with the *F* scores 0.54, 0.86, and 0.90, respectively. About 20% of tweets were classified as happy. Relatively few terms (less than 25) were necessary to characterize the majority of tweets on food and physical activity. Data from over 70,000 census tracts from the United States suggest that census tract factors like percentage African American and economic disadvantage were associated with lower census tract happiness. Urbanicity was related to higher frequency of fast food tweets. Greater numbers of fast food restaurants predicted higher frequency of fast food mentions. Surprisingly, fitness centers and nature parks were only modestly associated with higher frequency of physical activity tweets. Greater state-level happiness, positivity toward physical activity, and positivity toward healthy foods, assessed via tweets, were associated with lower all-cause mortality and prevalence of chronic conditions such as obesity and diabetes and lower physical inactivity and smoking, controlling for state median income, median age, and percentage white non-Hispanic.

**Conclusions:**

Machine learning algorithms can be built with relatively high accuracy to characterize sentiment, food, and physical activity mentions on social media. Such data can be utilized to construct neighborhood indicators consistently and cost effectively. Access to neighborhood data, in turn, can be leveraged to better understand neighborhood effects and address social determinants of health. We found that neighborhoods with social and economic disadvantage, high urbanicity, and more fast food restaurants may exhibit lower happiness and fewer healthy behaviors.

## Introduction

There is an increasing recognition that health is determined by a myriad of factors, including where you live, play, and work [1-5]. Poor access to healthy food [6-10], abundance of fast food chains [11], lack of recreational facilities [12,13], and higher crime rates [7,14] have been shown to predict higher obesity rates. Environmental exposure to toxins, noise, and violence can be detrimental to health [15,16]. Conversely, neighborhood resources such as playgrounds for children, grocery stores, and gyms can be beneficial to health [17]. Adverse neighborhood conditions converge in poor, minority neighborhoods [18-21], thereby increasing health disparities.

Social environments can offer social and emotional support that buffers stressful life events [22]. Johns and colleagues found that neighborhoods with higher social cohesion had lower posttraumatic stress disorder [23]. Higher community happiness levels are linked with lower obesity, hypertension, and suicide rates as well as increased life expectancy [24-29]. Evidence also suggests that emotional states such as happiness, optimism, depression, or suicidality can spread through social networks [30-33]. The social environment can offer opportunities for social control in regulating unhealthy behaviors and facilitating the social learning of healthy behaviors but can also promote risky behaviors. Health behaviors, such as food consumption, health screening, smoking, alcohol consumption, drug use, and sleep have also been observed to spread through social networks [34-37].

The extreme scarcity of neighborhood data greatly limits research on neighborhood effects. Some places [38,39] have extensive neighborhood data collected on them, but they are the anomaly rather than the rule, and it is difficult to make comparisons across geographies because available measures vary greatly across them. Neighborhood data collection is expensive and time consuming and only available for certain time periods [40]. Widespread usage of the Internet and open recording of many transactions (eg, Yelp reviews, Foursquare check-ins, and reporting of personal opinions and behaviors through social media) has led to the availability of massive amounts of data that enable understanding of previously hidden local area interactions. Researchers are increasingly utilizing social media and user-generated data to track health behaviors and perform health surveillance (eg, for outbreak detection) [41-45]. Others have used social media to track sleep issues [46], personal health status disclosed by Twitter users [47,48], and patient-perceived quality of care [49].

In this study, we explored the utility of building a national neighborhood database from geotagged Twitter data to characterize well-being and health behaviors. We validated our machine learning algorithm for constructing indicators of happiness, food, and physical activity by comparing machine-generated values to values generated by human labelers. In addition, we explored associations between Twitter-derived neighborhood variables and neighborhood demographic and economic characteristics. This project makes significant, relevant contributions to the field because neighborhood environments are increasingly linked to an array of important health outcomes and this project addresses the limits to research resulting from the lack of neighborhood data by providing new, cost-efficient data resources and methods for characterizing neighborhoods. To our knowledge, our study was the first to attempt to create a national neighborhood database from Twitter data, with indicators constructed for public health researchers. The only other type of neighborhood data that is consistently available for local areas is census data on the compositional characteristics of neighborhoods. Twitter is uniquely suited to characterize the social environment, including prevalent sentiment and health behaviors.

## Methods

### Social Media Data Collection

From February 2015 to March 2016, we utilized Twitter’s streaming application programming interface (API) to continuously collect a random 1% sample of publicly available tweets with latitude and longitude coordinates. Given that neighborhood researchers differ in their use and interest in data at the census tract and zip code level, we constructed neighborhood indicators at both levels thereby increasing the flexibility of our dataset to address the potential data needs of other researchers. In total, we collected 79,848,992 million geotagged tweets from 603,363 unique Twitter users in the contiguous United States (including District of Columbia). The median number of tweets per user was 4. Job postings (identified through hashtags #hiring, #jobs, and #job) were removed from the final analytic sample of tweets because these were pervasive and not central to the neighborhood variables we constructed.

### Spatial Join and Neighborhood Definition

Each geotagged tweet was assigned a corresponding census tract and zip code it falls within, based on the latitude and longitude coordinates of where the tweet was sent. This spatial join procedure was implemented in Python (version 2.7.12; Python Software Foundation), a popular programming language for spatial data processing [50]. Specifically, Python libraries were used to read shapefile format vector data (PyShp 1.1.4), build an R-tree index on the polygon data (Rtree 0.8.2), and perform a spatial join operation (Shapely 1.5.12 and Fiona 1.6.1). The R-Tree was used to build a spatial index [51] on the national census tract and zip code polygon data to speed computation. Tweets that were not assigned a census tract or zip code location included those with destinations bordering the United States (ie, Mexico and Canada). We linked 99.8% of tweets with geocordinates to their respective 2010 census tract and zip code locations. The term *neighborhood* used in this paper refers to both zip codes and census tracts. We mapped tweets to these two geographic boundaries because they are among the most popular neighborhood definitions utilized by public health researchers [52-54].

### Processing Tweets

Duplicate tweets (ie, tweets with the same tweet ID, <1%) were removed computationally. Although Twitter’s API collects a random subset of 1% of publicly available tweets, users (especially spam accounts) who tweet often have potentially greater influence on variable values we construct. We examined outliers in our datasets (defined as the users whose tweets accounted for more than 1% of tweets in our dataset) and eliminated automated accounts and accounts for which the majority of tweets were advertisements. Processing and statistical analysis tasks were performed with Stata MP13 (StataCorp LP).

### Construction of Neighborhood Variables From Twitter Data

From geotagged tweets, we derived variables that characterize happiness, food, and physical activity. Each tweet was divided into tokens using the Stanford tokenizer [55]. For processing of English text, tokens roughly correspond to words. We then built various algorithms utilizing tokens to create variables that characterize happiness and make references to food and physical activity. Below we describe in more detail our algorithms.

### Sentiment Analysis

To conduct sentiment analysis, we utilized the Machine Learning for Language Toolkit (MALLET; AK McCallum, 2002), a Java-based package for statistical natural language processing, document classification, clustering, topic modeling, information extraction, and other machine learning applications to text. We leveraged the Maximum Entropy text classifier in MALLET to classify tweets as happy and not happy [56]. In order to train our classifier, we obtained training sets from the following resources: Sentiment140 [57], Sanders Analytics [58], and Kaggle [59]. We trained our classifier to differentiate between happy and not happy sentiments. We then ran our classifier on our national Twitter data to compute a happy score (range 0-1) for each tweet, where higher happiness scores indicate more positive sentiment. MALLET estimates predicted probabilities that a tweet is happy based upon word-level features. The classifier uses search-based optimization to assign weights that maximize the likelihood of the training data. However, unlike Naïve Bayes, the Maximum Entropy classifier does not assume conditional independence among features.

To calibrate the generated happiness scores with human generated labels, two raters manually read a random subset of 1200 tweets and assigned a value of 1 to happy tweets and 0 to not happy tweets. The initial interrater reliability was 92%, and discordant values were reviewed until a 100% agreement between raters was reached. To decide on a cut point for MALLET scores at which we would classify tweets as happy, we computed accuracy levels at different cut points of MALLET scores (Multimedia Appendix 1). Increasing the MALLET score improves the accuracy against human annotations but also reduces the calculated prevalence of tweets deemed as happy. A MALLET score of 0.80 achieves the highest level of accuracy while still maintaining a prevalence of happy tweets of 19% (which approximates the prevalence obtained by human annotations). Area under the receiver operating characteristic curve is approximately 0.7 for all MALLET cut points between 60 and 85.

### Food Analysis

We compiled a list of over 1430 popular food words from the US Department of Agriculture’s National Nutrient Database [60]. Each food item was associated with a measure of caloric density, operationalized as calories per 100 grams. Fruits, vegetables, nuts, and lean proteins (ie, fish, chicken, and turkey) were labeled as healthy foods (340 food terms in total). Fried foods were not considered healthy foods. Our food list also contained popular national fast food restaurants such as McDonald’s and Kentucky Fried Chicken (captured via 154 food terms including popular variations of restaurant names) to enable quantification of fast food references. From April 2015 to March 2016, we collected and processed 4,041,521 geotagged food tweets. In the food dataset, the median number of tweets per user was 12 tweets.

To analyze food culture, each tweet was examined for words or phrases matching those on our list. Each food item on our list was described by one or two words. Our text-matching algorithm first searched over a tweet for matches to two-word foods (eg, orange chicken). It then searched over the remaining words for matches to one-word food terms (eg, taco). We computed caloric density by summing up all the foods mentioned in the tweet. We also created a count of healthy food references and fast food restaurant references for each tweet. Moreover, we leveraged our sentiment analysis to assess sentiment toward food. Specifically, we tracked sentiment around healthy foods and fast food. These variables (any food references, healthy food references, fast food references, caloric density, and sentiment toward healthy foods and fast food) were then aggregated and summarized at the census tract and zip code level to create neighborhood indicators of food culture.

### Physical Activity Analysis

We created a list of physical activities using published lists of physical activity terms gathered from physical activity questionnaires, compendia of physical activities, and popularly available fitness programs [61,62]. Our physical activity list had 376 different activities that incorporate gym-related exercise (eg, treadmill, weight lifting), sports (eg, baseball), recreation (eg, hiking, scuba diving) and household chores (eg, gardening). We excluded popular phrases that generally do not relate to physical activity such as “walk away” and “running late.” Using metabolic equivalents associated with physical activities, we quantified the exercise intensity of each physical activity mention, scaled for a duration of 30 minutes and for a 155-pound individual [63], which approximates the weight of an average American adult [64,65].

Upon piloting our algorithm, we identified commonly used phrases or pop culture references that do not involve physical activity (eg, walking dead) which were manually coded and excluded. Moreover, in order to help reduce the possibility that the tweet was about watching rather than actually participating in the physical activity, we excluded the tweet if it contained any of the following terms: “watch,” “watching,” “watches,” “watched,” “attend,” “attending,” “attends,” and “attended.” In reviewing preliminary labeled physical activity data, we found that most tweets (over 90%) pertaining to team sports (eg, baseball, basketball, football, soccer) were about watching games rather than participating in them. Thus, for team sports, we required that the tweet include the words “play,” “playing,” or “played.”

Our algorithm created the following physical activity variables for each tweet: any physical activity mention, exercise intensity, and sentiment around physical activity. From April 2015 to March 2016, we collected 1,473,976 geotagged physical activity tweets. In the physical activity dataset, the median number of tweets per user was 5 tweets.

### Quality Control Activities

A total of 5000 tweets have been manually labeled by two of the authors for quality control activities on food and physical activity. The authors manually labeled whether each tweet was food-related (2000), non–food-related (500), physical activity-related (2000), or non–physical activity-related (500). Excellent interrater reliability was achieved with greater than 90% agreement in all categories, and differences were discussed and resolved.

Among tweets our algorithm had labeled as food-related, 83% were labeled accurately when compared to labels generated by manual categorization. Among tweets our algorithm had labeled as non–food-related, 81% were labeled accurately (ie, both algorithm and human categorizers labeled the tweet as non–food-related). Overall, accuracy for food tweets was 83% and the *F* score was 0.86. It should be noted our algorithm could label a food-related tweet as non–food-related if the food reference was not in our food dictionary. Food items that are often associated with non-related food meaning, such as “perch,” have been excluded from our food dictionary. For tweets that had been mislabeled as food-related, commons reasons included food term used as a metaphor, in a pun, or for food advertisement.

Among tweets our algorithm had labeled as physical activity-related, 82% of them were labeled accurately when compared to labels generated by human categorizers. An accuracy of 97% was found among tweets labeled as non-physical activity-related by our algorithm. The *F* score was 0.90 and the overall accuracy was 85% for physical activity tweets. Typical errors in classification of physical activity tweets included the use of an idiom (eg, running late) or the tweet was about watching sports games rather than playing sports.

Additionally, we evaluated our algorithm on its ability to identify relevant food and physical activity terms within tweets. To do this, we examined a random subset of tweets that the algorithm had identified as positive for food (n=200) and physical activity (n=200). Here we focused on the accuracy of our algorithm to conduct string detection. We manually read the tweets to verify that manual annotations agreed with the terms detected. For food tweets, 87% of manual annotations matched all detected terms from the algorithm. Errors for nondetection of terms occurred when the tweet included a hashtag that had multiple food terms without spacing (eg, #chocolatebrownie) or when there were misspellings (eg, sandwhich) or when the food was not on the food list. String detection for physical activity-related terms was more accurate with 98% of manual annotations matching detected terms from the algorithm. Errors included the omission of certain terms from the dictionary (eg, cycling) and use of hashtags without spacing of terms (#runrunrun).

We further evaluated our sentiment analysis activities through Amazon Mechanical Turk (Mturk; Amazon.com Inc, Seattle, WA, USA), an online crowdsourcing marketplace [66]. We randomly selected 500 tweets with 50% labeled as happy and 50% as not happy by our algorithm. Then, we created 20 online surveys through random sorting, with each survey consisting of 25 tweets. We asked participants to rate the sentiment of each tweet. All 20 surveys were live on April 1, 2015. Each online survey closed itself when 15 responses had been reached; the last survey closed on April 5, 2015. For each completed survey, 25 cents ($0.25) was deposited into the participant’s Mturk account. A total of 32 participants completed 300 surveys (ie, 15 responses per survey, 20 surveys). Some participants completed multiple surveys rather than just one. Each tweet was then assigned a label of either happy or not happy based on the modal response from Mturkers (participants from Amazon Mturk). We found an accuracy of 69% for happy tweets and 80% for nonhappy tweets when compared to responses from Mturkers. The overall accuracy for sentiment was 78%, with an *F* score of 0.54.

We additionally compared performance of MALLET with two other sentiment analysis techniques: a popular bag-of-words technique involving the use of a 10,000 word list [67] and Sentiment140, a machine-learning classifier [68]. Among the 500 control tweets from our LabMT experiment, the bag-of-words algorithm had an accuracy of 73% (*F* score 0.55) and Sentiment140 was had an accuracy of 77% (*F* score 0.47).

### Other Publicly Available Neighborhood Data

To examine how Twitter-derived neighborhood variables relate to more traditional neighborhood variables, we merged our social media dataset with the 2010 Census and 2014 American Community Survey data which comprised the following demographic, household, and economic characteristics: household size, median family income and percent of the following: 65 years and older age group, 10-24 years, male, African American, white, Hispanic, households with relatives (other than spouse and children), households with unmarried partner, single female-headed households, householder living alone, owner-occupied housing, college graduates, unemployed, less than a high school degree and families living in poverty. A census tract was urban if the geographic centroid of the tract was in an area with more than 2500 people; all other tracts are rural. A zip code was defined as urban if the majority (75% or more) of its land area was characterized as urban (ie, containing at least 2500 people).

Data on business types at the zip code level were obtained from the 2013 US Census Bureau zip code business patterns accessed via American FactFinder [69]. The following North American Industry Classification System (NAICS) codes were utilized to categorize businesses: 722410 (drinking places [alcoholic beverages]; these places are also known as bars, taverns, night clubs and primarily serve alcohol and may have limited food services) and 722511 (full-service restaurants; these include, for instance, diners and steakhouses). Fast food was defined by the following NAICS codes: 722513 (limited-service restaurants; these include carryout restaurants, drive-in restaurants, and other fast food restaurants) and 722515 (snack and nonalcoholic beverage bars). We also tracked supermarkets and grocery stores (NAICS code 445110) and convenience stores (NAICS code 445120). To examine associations between Twitter physical activity mentions and presence of recreational facilities, we retrieved business data for the following types of establishments: fitness and recreational sports centers (NAICS code 713940), nature parks (NAICS code 712190), zoos and botanical gardens (NAICS code 712130), golf courses and country clubs (NAICS code 713910), skiing facilities (NAICS code 713920), and bowling centers (NAICS code 713950).

We obtained state-level health outcome data including age-adjusted death rates due to all-causes and homicides from 2013 National Vital Statistics Reports. Data in this report was based on information from all resident death certificates filed in the 50 states and the District of Columbia. Death certificates are generally completed by funeral directors, attending physicians, medical examiners, and coroners. Age-adjusted death rates expressed per 100,000 population were based on the 2000 US standard population. Causes of death statistics were classified by the International Classification of Diseases, Tenth Revision, and based on the underlying cause of death.

We obtained age-adjusted prevalences of health risk behaviors and chronic conditions of US adult residents for the 50 states from the 2013 Behavioral Risk Factor Surveillance System (BRFSS), the nation's premier system of health-related telephone surveys. The questionnaires were created by BRFSS state coordinators and Centers for Disease Control and Prevention staff. BRFSS data includes self-reported physical activity, self-rated health, body mass index (BMI, kg/m^2^), and medical diagnoses of chronic conditions aggregated to the state level. Data from a national health survey suggests that BMI estimates derived from self-reported height and weight were lower than those are derived from measured height and weight, although BMI differences were generally less than 1.0 kg/m^2^ across sex and age groups [70]. State-level BRFSS data is publicly available. Smaller area aggregations can require data use agreements. In addition to state-level BRFSS data, we also utilized restricted-access zip-code–level data from the 2009-2014 Utah BRFSS survey to examine zip-code–level health outcomes [71,72].

### Regression Analyses

We implemented adjusted linear regression models to examine associations between area-level Twitter characteristics and other area-level characteristics (demographics, business characteristics, and health outcomes). To facilitate interpretation of findings for different variables, we standardized all variables to have a mean of zero and standard deviation of one. We investigated spatial autocorrelation and found that Moran’s I was highest for census tract Twitter happiness (0.12) and less than 0.04 for other Twitter tract and zip code summaries. To account for spatial autocorrelation of area-level values in linear regression analyses, we adjusted standard errors for clustering of census tract and zip code values within a county. Statistical analyses were implemented with Stata MP13 (StataCorp LP) and ArcGIS Desktop version 10.1-10.3 (Esri).

## Results

Table 1 displays descriptive statistics. Approximately 20% of tweets were happy. About 5.1% of tweets were about food and 1.8% were about physical activity. The mean and median caloric density of food references were 239 and 209 calories per 100 grams, respectively. Tweets about healthy food were happier than tweets about fast food (28.3% vs 14.5%; *P*<.001). The mean and median exercise intensity of physical activity mentions (assuming 30 minutes for a 155-pound person) were 199 and 130 calories, respectively.

**Figure 1:**
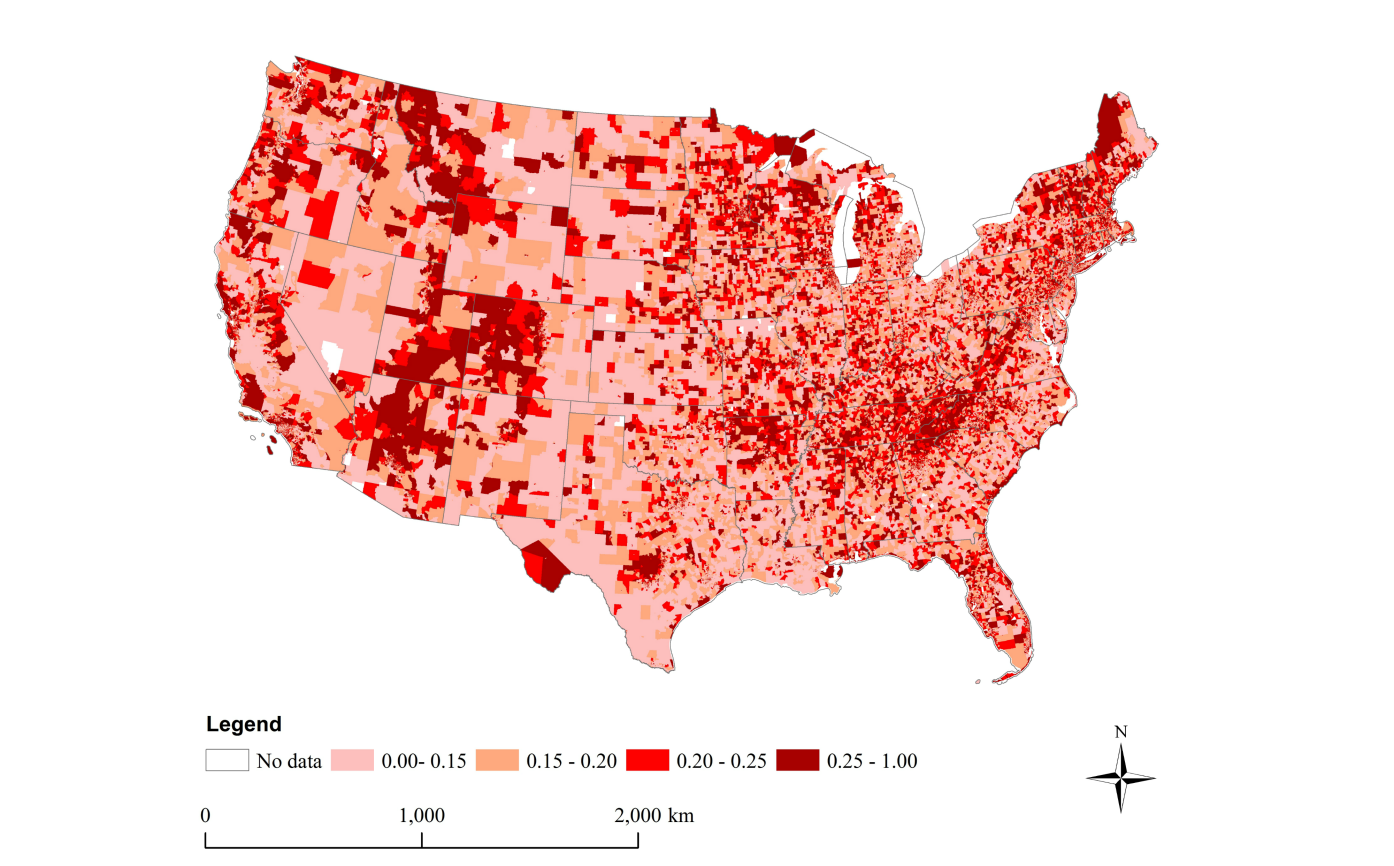
National distribution of happy tweets, by census tract. Geotagged tweets were spatially joined to their 2010 census tract locations and sentiment scores were computed. This color coded map presents the proportion of happy tweets in each census tract, with darker colors signifying higher proportions of happy tweets.

Figure 1 presents the spatial distribution of happy tweets by census tract, highlighting variation across the United States. Multimedia Appendix 2 presents the spatial distribution of happy tweets by zip code. The proportion of happy tweets was highest in the following states: Montana, Tennessee, Utah, New Hampshire, Arkansas, Maine, Colorado, and New York (Multimedia Appendix 3). By contrast, the proportions of happy tweets were lowest for the following states: Louisiana, North Dakota, Oregon, Maryland, Texas, Delaware, West Virginia, and Ohio (Multimedia Appendix 3).

Table 2 presents the results of adjusted linear regression analyses examining the associations between population characteristics and Twitter-derived characteristics at the census tract level (percent of tweets that were happy, percent of tweets about healthy food, percent of tweets about fast food, and percent of tweets about physical activity). Census tract characteristics like percent African American (beta coefficient, B=−.11), greater household size (B=−.18), and economic disadvantage (B=−.19) were related to lower tract happiness. Economic disadvantage was negatively related to healthy food tweets (B=−.09), fast food tweets (B=−.09), and physical activity tweets (B=−.03). Urbanicity was strongly related to higher frequency of fast food tweets (B=.29). Greater household size was related to both lower healthy food tweets (B=−.11) and fast food tweets (B=−.07).

**Table 1:** Descriptive statistics of our national Twitter database, April 2015 to March 2016 (N=79,848,992).

		Mean (SD)
**Happiness**		
	% Tweets that are happy	19.9 (6.7)
**Food culture**		
	% Tweets about food	5.1 (22.0)
	% Food tweets about healthy foods	15.9 (36.6)
	% Food tweets about fast food	9.2 (29.0)
	Caloric density of food tweets (per 100 grams)	238.5 (219.8)
	% Food tweets that are happy	27.0 (44.4)
	% Healthy food tweets that are happy	28.3 (45.0)
	% Fast food tweets that are happy	14.5 (35.2)
**Physical activity culture**		
	% Tweets about physical activity	1.8 (13.3)
	Exercise intensity (per 30 minutes)	199.1 (117.5)
	% Physical activity tweets that are happy	28.2 (45.0)

**Table 2:** Demographic and economic predictors of happy, food, and physical activity tweets from 70,515 census tracts (data source: 2010 US Census data).

Tract characteristics	% happy tweets Beta (95% CI)^a^	*P* value	% healthy food tweets Beta (95% CI)^a^	*P* value	% fast food tweets Beta (95% CI)^a^	*P* value	% physical activity tweets Beta (95% CI)^a^	*P* value
Urban (yes)	−.01 (−.04 to .03)	.79	.01 (−.02 to .03)	.54	.29 (.26 to .31)	<.001	−.02 (−.03 to −.01)	<.001
Population density	.06 (.03 to .08)	<.001	.04 (.02 to .07)	.001	−.03 (−.03 to −.02)	<.001	.00 (−.01 to .00)	.82
% 65 years and older	.02 (−.01 to .04)	.09	−.03 (−.04 to −.02)	<.001	−.03 (−.04 to −.01)	<.001	.02 (.02 to .03)	<.001
% 10-24 years	−.02 (−.04 to .00)	.01	−.05 (−.05 to −.04)	<.001	.00 (−.01 to .01)	.49	.00 (−.01 to .00)	.14
% Male	.04 (.03 to .06)	<.001	.01 (.00 to .02)	.21	−.05 (−.06 to −.04)	<.001	.01 (.01 to .02)	<.001
% African American	−.11 (−.14 to −.07)	<.001	−.03 (−.04 to −.01)	<.001	−.03 (−.04 to −.02)	<.001	−.01 (−.02 to −.01)	<.001
% Hispanic	−.04 (−.08 to .00)	.05	.02 (.01 to .03)	.00	.07 (.05 to .09)	<.001	.00 (.00 to .00)	.77
Household size	−.18 (−.20 to −.15)	<.001	−.11 (−.12 to −.09)	<.001	−.07 (−.09 to −.05)	<.001	−.01 (−.01 to −.01)	<.001
Economic disadvantage^b^	−.19 (−.21 to −.16)	<.001	−.09 (−.10 to −.08)	<.001	−.09 (−.10 to −.07)	<.001	−.03 (−.04 to −.03)	<.001

^a^Adjusted linear regression included all tract demographic and economic predictors simultaneously. Standard errors accounted for clustering at the county level.

^b^Economic disadvantage factor score derived from the following census tract characteristics: percent female-headed households, percent families living in poverty, unemployment rate, percent college graduates (reverse coded), and median family income (reverse coded).

Sensitivity analyses were performed to examine the relationship between population characteristics and happiness for a different unit of aggregation: zip code areas. Relationships seen at the census tract level were similar to those at the zip code level, although they were more muted at the zip code level (not shown). This may be the case because census tracts are designed to be relatively homogenous with regard to characteristics such as economic status and demographic characteristics [73].

**Figure 2:**
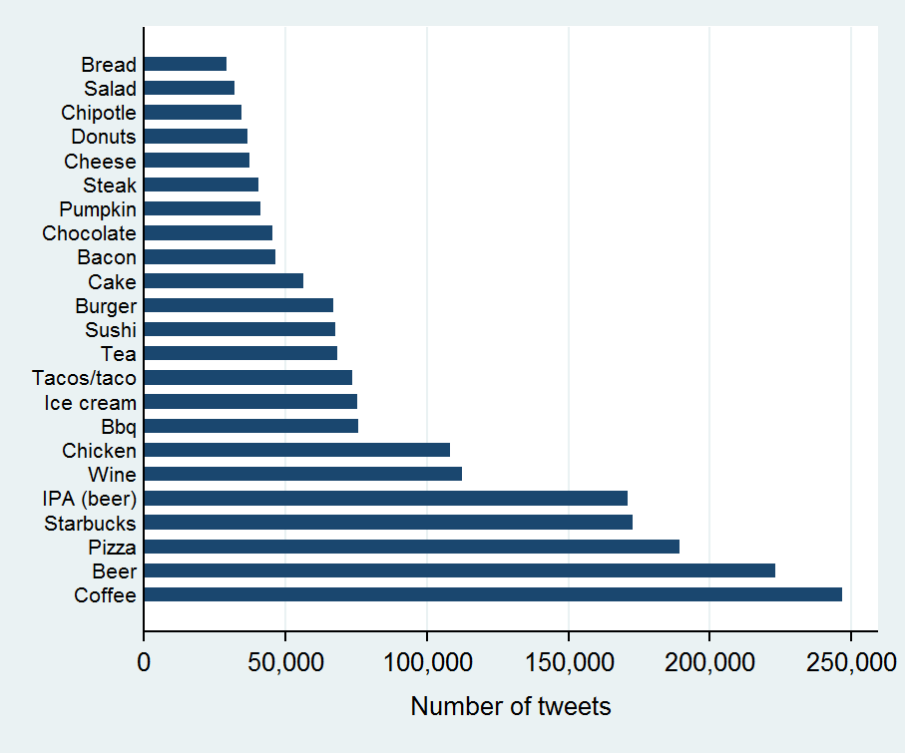
Items in the top 50% of food tweets.

**Figure 3:**
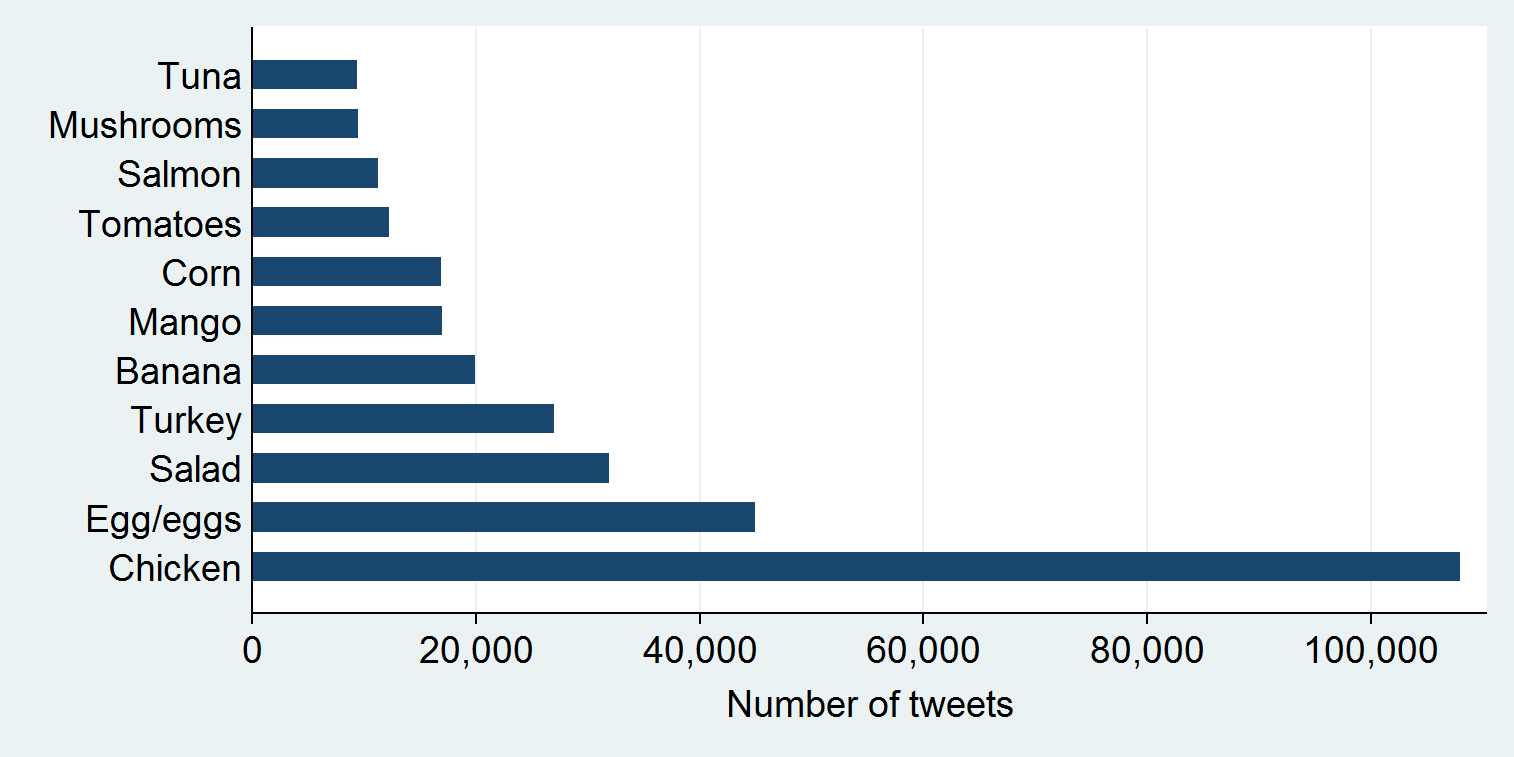
Items in the top 50% of healthy food tweets.

Healthy foods (ie, vegetables, fruits, nuts, lean proteins) composed 15.9% of food tweets, while fast food restaurant mentions composed 9.2% of food tweets. The most popular foods include coffee, beer, pizza, wine, chicken, ice cream, and sushi (Figure 2). Popular healthy food terms included chicken, eggs, salad, turkey, and banana (Figure 3). Starbucks was the most popular fast food place mentioned (accounting for 46% of all fast food restaurant mentions), followed by Chipotle (9.2%), Taco Bell (5.4%), and Buffalo Wild Wings (5.2%). We additionally examined the relationship between food tweets and business characteristics. At the zip code level, greater numbers of fast food restaurants were associated with more fast food tweets (B=.15), and higher caloric density of food mentions (B=.08). Urban areas had tweets with higher caloric density (B=.08) and more fast food restaurant mentions (B=.16). Happy tweets were more prevalent in zip codes with higher numbers of businesses (B=.11) and full-service restaurants (B=.16). Higher numbers of fast food restaurant (B=−.16) and convenience stores (B=−.07) were related to fewer happy tweets (Table 3).

**Figure 4:**
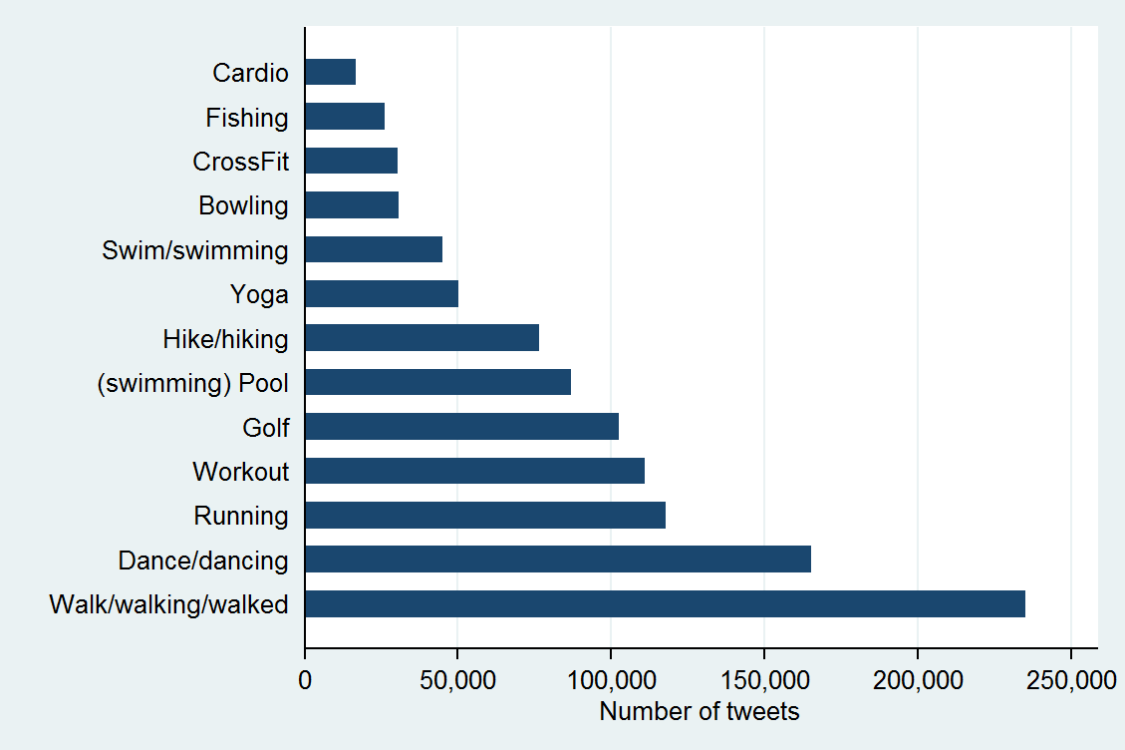
Items in the top 75% of physical activity tweets.

Additionally, relatively few physical activity terms (13 terms) accounted for 75% of physical activity tweets (Figure 4) although our data collection system was set up to collect tweets on 376 physical activity terms. The most popular terms included walking, dancing, and running. At the zip code level, greater numbers of fitness and recreational sports centers were related to higher exercise intensity (B=.05) and happier tweets (B=.07). Surprisingly, the presence of nature parks was not associated with physical activity mentions. Urbanicity was associated with lower frequency of physical activity tweets and happy tweets but higher exercise intensity (Table 4). In supplemental analyses, we examined information on number of miles covered during physical activity if that was mentioned in the tweet (n=36,291; median 3.1 miles). Even fewer tweets contained information on amount of time the person engaged in physical activity. Among 5823 tweets that mentioned hour(s) of physical activity, the median amount was 2 hours. Among 2402 tweets that only referred to minutes of physical activity, the median number of minutes was 20.

**Table 3:** Zip code and business characteristics as predictors of food tweets and happiness (data sources: 2013 zip code business patterns and 2010 US Census data).

Zip code characteristics	Average caloric density of food tweets n=21,756 Beta (95% CI)^a^	*P* value	% fast food tweets n=21,756 Beta (95% CI)^a^	*P* value	% happy tweets n=26,584 Beta (95% CI)^a^	*P* value
Urban (yes)	.08 (.05 to .11)	<.001	.16 (.12 to .20)	<.001	−.02 (−.06 to .02)	.29
Population density	.00 (.00 to .01)	.24	.00 (−.01 to .01)	.86	.01 (.00 to .03)	.18
Number of businesses	−.01 (−.02 to .01)	.34	.02 (.00 to .04)	.04	.11 (.08 to .15)	<.001
Businesses that sell alcohol	−.03 (−.04 to −.02)	<.001	−.04 (−.05 to −.04)	<.001	−.01 (−.02 to .00)	.02
Full service restaurants	−.04 (−.06 to −.02)	<.001	.01 (−.01 to .03)	.43	.16 (.13 to .20)	<.001
Fast food restaurants	.08 (.06 to .10)	<.001	.15 (.13 to .17)	<.001	−.16 (−.20 to −.12)	<.001
Grocery stores	.01 (.00 to .01)	.28	−.04 (−.05 to −.03)	<.001	−.02 (−.04 to .00)	.05
Convenience stores	.02 (.01 to .02)	<.001	−.03 (−.04 to −.02)	<.001	−.07 (−.08 to −.05)	<.001

^a^Adjusted linear regression included all zip code and business characteristics simultaneously. Standard errors accounted for clustering at the county level.

**Table 4:** Zip code and business characteristics as predictors of physical activity tweets and happiness (data sources: 2013 zip code business patterns and 2010 US Census data).

Zip code characteristics	% physical activity tweets n=26,839 Beta (95% CI)^a^	*P* value	Exercise intensity n=20,715 Beta (95% CI)^a^	*P* value	% happy tweets n=26,839 Beta (95% CI)^a^	*P* value
Urban (yes)	−.09 (−.11 to −.07)	<.001	.07 (.04 to .11)	<.001	−.08 (−.12 to −.04)	<.001
Population density	−.01 (−.02 to .00)	.01	−.01 (−.01 to .00)	.03	.01 (.00 to .02)	.08
Fitness/recreational centers	.01 (.00 to .02)	.003	.05 (.04 to .06)	<.001	.07 (.06 to .08)	<.001
Nature parks	.01 (.00 to .02)	.05	−.01 (−.01 to .00)	.21	.03 (.02 to .04)	<.001
Zoos/botanical gardens	.00 (.00 to .01)	.19	.00 (−.01 to .00)	.35	.02 (.01 to .03)	<.001
Golf/country clubs	.03 (.02 to .03)	<.001	−.05 (−.06 to −.04)	<.001	.03 (.02 to .04)	<.001
Skiing facilities	.04 (.04 to .05)	<.001	.02 (.02 to .03)	<.001	.03 (.02 to .03)	<.001
Bowling centers	−.01 (−.02 to −.01)	<.001	−.01 (−.02 to .00)	.01	−.02 (−.03 to −.01)	<.001

^a^Adjusted linear regression included all zip code and business characteristics simultaneously. Standard errors accounted for clustering at county level.

**Table 5:** Twitter happiness as a predictor of health outcomes in 232 zip codes in Utah (data source: Utah Behavioral Risk Factor Surveillance System [BRFSS] survey 2009-2014. BRFSS underwent design feature changes. Life dissatisfaction values were only available for 2009 and 2010. All other variables were averages from available data from 2011-2014).

Zip code health outcomes	Beta (95% CI)^a^ n=232	*P* value
Life dissatisfaction	.01 (−.13 to .15)	.91
Self-rated health (higher score=worse health)	−.08 (−.21 to .05)	.21
Any past month physical activity/exercise	.13 (.00 to .26)	.05
Body mass index (kg/m^2^)	−.13 (−.26 to −.01)	.04

^a^Separate linear regression models for each zip code health outcome.

**Table 6:** State level Twitter sentiment predictors of health outcomes (N=49 states in the contiguous United States plus District of Columbia. Data sources: 2013 National Vital Statistics Reports and 2013 Behavioral Risk Factor Surveillance System [BRFSS] survey on adults).

	Twitter predictor variables					
State-level adult health outcomes	Happiness Beta (95% CI)^a^	*P* value	Positive sentiment toward healthy foods Beta (95% CI)^a^	*P* value	Positive sentiment toward physical activity Beta (95% CI)^a^	*P* value
All-cause mortality per 100,000	−32.34 (−61.59 to −3.09)	.03	−23.51 (−40.54 to −6.48)	.01	−25.37 (−42.00 to −8.74)	.004
Homicide per 100,000	−1.02 (−1.98 to −.06)	.03	−.76 (−1.28 to −.25)	.01	−.75 (−1.28 to −.23)	.01
% With diabetes	−.58 (−1.05 to −.12)	.02	−.52 (−.78 to −.27)	<.001	−.41 (−.68 to −.14)	.004
% With obesity	−2.27 (−3.35 to −1.18)	<.001	−1.67 (−2.25 to −1.09)	<.001	−1.43 (−2.05 to −.80)	<.001
% Poor/fair self-rated health	−1.13 (−2.13 to −.13)	.03	−.77 (−1.36 to −.19)	.01	−.61 (−1.21 to −.02)	.05
% With high cholesterol	−.78 (−1.66 to .11)	.08	−.51 (−1.04 to .01)	.06	−.75 (−1.25 to −.26)	.003
% Physical inactivity	−2.46 (−4.80 to −.12)	.04	−2.32 (−3.61 to −1.03)	.001	−1.59 (−2.97 to −.22)	.02
% Current smoking	−1.47 (−2.68 to −.27)	.02	−1.20 (−1.88 to −.52)	.001	−1.14 (−1.82 to −.45)	.002

^a^Each cell in the table represents the coefficient estimate of the predictor variable (given by the column) on the state-level health outcome (given by the row). Adjusted linear regression models controlled for state-level demographics: median age, % non-Hispanic white, median household income.

Additionally, merging in health-related datasets, we examined associations between our Twitter-based variables and other measures of health and well-being. Utilizing data from the 2009-2014 BRFSS in Utah, we found that zip codes in Utah with higher Twitter happiness scores were associated with lower body mass index and higher physical activity (Table 5). However, Twitter happiness scores were not statistically significantly related to self-rated health or life satisfaction.

Greater state-level happiness, as indicated by tweets, was related to lower prevalence of obesity; a one standard deviation increase in happiness was associated with two percentage points lower prevalence in obesity. Greater positive sentiment for healthy foods was related to lower prevalence of diabetes and obesity and lower percent of the population who are physically inactive or current smokers (Table 6). Positive sentiment toward physical activity was related to lower obesity.

Table 7 presents adjusted regression results for additional Twitter-derived variables (percentage of food tweets about healthy foods, percentage of food tweets about fast food, and percentage of tweets about physical activity) and a select number of state health outcomes. Out of the three Twitter-derived variables, percentage of tweets about physical activity was the strongest and most consistent predictor; more online discussion about physical activity was related to lower all-cause mortality and lower prevalence of obesity and fair/poor self-rated health.

**Table 7:** State level Twitter food and physical activity characteristics as predictors of health outcomes (N=49 states in the contiguous United States plus District of Columbia. Data sources: 2013 National Vital Statistics Reports and 2013 Behavioral Risk Factor Surveillance System [BRFSS] survey on adults).

	State-level adult health outcomes					
Twitter predictors	All-cause mortality per 100,000 Beta (95% CI)^a^	*P* value	% with obesity Beta (95% CI)^a^	*P* value	% poor/fair self-rated health Beta (95% CI)^a^	*P* value
% Of food tweets about healthy food	11.74 (−6.48 to 29.96)	.20	−.09 (−.64 to .45)	.73	.11 (−.48 to .70)	.71
% Of food tweets about fast food	9.84 (−8.56 to 28.25)	.29	.68 (.13 to 1.23)	.02	.77 (.18 to 1.37)	.01
% Of tweets about physical activity	−28.17 (−46.68 to −9.65)	.004	−1.86 (−2.41 to −1.31)	<.001	−.89 (−1.49 to −.29)	.01

^a^Adjusted linear regression models were run separately for each state-level health outcome (column) and included all three predictors (row) simultaneously in addition to the following state-level control variables: median age, % non-Hispanic white, median household income. Beta coefficient represents a change in the outcome for every standard deviation change in the predictor (row variable).

## Discussion

### Principal Findings

In this paper, we detail the building of a new national neighborhood data repository constructed from Twitter data which addresses a pressing need for neighborhood data that are available across large geographies and can be updated efficiently and cost-effectively. We demonstrate that simple machine learning algorithms for the construction of indicators for happiness, food, and physical activity can agree extremely well with manually generated labels. About one-fifth of tweets were identified as happy. There was substantial spatial variation in happiness across the United States. For instance, the proportion of tweets that were happy in Montana (the most happy state) was 10% greater than in Louisiana (the least happy state). Only a few terms are needed to capture the majority of tweets on food and physical activity. Economic disadvantage, urbanicity, and presence of fast food restaurants predicted lower area level happiness and lower frequency of healthy behavior mentions on Twitter. Moreover, we find that Twitter area-level characteristics are correlated with area-level health outcomes relating to health behaviors, chronic diseases, mortality, and self-rated health.

### Study Findings in Context

Social media represents an important new data resource that is increasingly being harnessed for public health efforts such as surveillance of smoking behavior and sentiment toward tobacco products [74]. However, few studies are leveraging social media data for the investigation of local area characteristics. More commonly, studies utilizing social media data examine patterns at the city, county, or state level [67,75] rather than at finer levels of aggregation, which is necessary for understanding the potential impacts of neighborhood conditions.

Neighborhoods can impact health through a myriad of pathways. Disadvantaged neighborhoods may have fewer resources that support physical activity and healthy diets. Poor and minority neighborhoods have fewer large supermarkets (where healthy foods are more abundant and affordable) compared to wealthy and majority white neighborhoods. Studies have documented increased fruit and vegetable consumption with more supermarket availability [17]. Poor neighborhoods, which have been labeled food deserts, also tend to have more fast food restaurants, which can contribute to weight gain [6]. In this study, we found that higher numbers of fast food restaurants were associated with higher frequency of fast food mentions, lower frequency of healthy food mentions, and less positive sentiment about healthy foods on Twitter. Our results align with a recent study conducted analyzing Instagram posts, which found that posts originating from census tracts deemed as food deserts contained fewer mentions of fruits and vegetables compared to Instagram posts outside food deserts [76]. Additionally, neighborhoods may promote poor health through psychosocial pathways. Living in neighborhoods that are unclean, noisy, and violent can be psychologically harmful through over-activation of the stress response [77,78].

We found that economic disadvantage was related to lower frequency of happy tweets. Previous research by Mitchell and colleagues found that higher socioeconomic status was associated with higher Twitter happiness scores at the city level. Moreover, they identified mild correlations (r=−0.34) between happiness and obesity rates for 190 metropolitan statistical areas [67] and that Twitter happiness scores were moderately correlated with other state-level indicators of well-being including shootings, the Peace index, America’s Health Ranking, and the Gallup-Healthways Well-Being Index (correlations ranged between 0.51 and 0.64) [67].

### Study Strengths and Limitations

In this paper, we describe the creation of a new neighborhood data repository constructed from Twitter data and merged with publicly available administrative datasets. However, this study is subject to several limitations. For instance, users of social media tend to be younger; in 2014, 37% of individuals aged 18 to 29 years old used Twitter compared to 12% of individuals aged 50 to 64 years and 10% among those 65 years and older. Nonetheless, adoption rates of social media have been steadily increasing [79]. Tweets also include information rarely found in other neighborhood sources. Twitter users are composed of individuals as well as groups of individuals, organizations, companies, and news outlets. Thus, compiling such information may allow for a more comprehensive examination of the social environment.

Moreover, we are only collecting a subset of publicly available tweets, and thus conclusions from our analytic sample may not generalize to the full population of tweets [80]. Our construction of neighborhood indicators from Twitter data necessitated that we restricted our data collection to geolocated tweets. We utilized Twitter’s API which allows the retrieval of a maximum resulting volume of 1% of the total tweets at any given time point. Previous studies suggest that about 1% to 2% of tweets may contain global positioning system location information [81,82] and that use of Twitter’s streaming API may obtain 40% to 90% of all geotagged tweets [81,82]. Tweets with location information may be different from those without. For example, tweets in which users share their locations may be more likely to contain public and social activities such as friends tweeting from a restaurant or an event. However, in sensitivity analyses with a subset of control tweets (n=138,152 tweets) collected from July 9 to July 14, 2015, we did not detect any statistically significant differences in happiness scores between tweets with and without geographic coordinates (not shown).

In creating our neighborhood indicators from Twitter data, we prioritized transparency and ease of implementation so that other researchers can replicate our algorithms. Our sentiment algorithm was trained to differentiate between happy and not happy sentiments (which encompasses neutral and sad sentiments). Thus, we were not able to specifically examine the prevalence of sad tweets, which may provide additionally useful information about the well-being of communities. In future work, we plan to target the identification of sadness. Our algorithms for food and physical activity implemented corpus-based classification with steps that are easily understandable. However, this technique does not take into account the entire context of sarcasm or humor in a tweet, challenges which still evade most natural language processing algorithms though some studies show promising results [83,84]. Our analysis of caloric density of food assumed calories per 100 grams. Most tweets do not specify the exact amount of food consumed, and thus our estimate is just an approximation.

Additionally, the content of tweets reflects the type of information that people feel comfortable reporting and may not represent the true spectrum of their feelings or their experiences. For instance, people may feel most comfortable presenting a neutral stance rather than voicing polarizing viewpoints. Certain foods (cupcakes) may get tweeted more often than others (celery). Additionally, we cannot be certain that the food that was tweeted was indeed consumed. Similarly, physical activity tweets may reflect a mixture of intentions, plans, and actual engagement in those physical activities. Also, exercise intensity for physical activities was assessed for 30 minutes of physical activity for an individual weighing 155 pounds, which can be an under- or overestimation depending on the type of activity and persons engaged in that activity.

### Conclusions

The epidemic rise in obesity and related chronic diseases in recent decades signal the importance of structural forces and social processes, but the dearth of data on contextual factors limits the investigation of multilevel effects on health. Social media data can be uniquely harnessed to capture social and cultural processes with potential impacts on health [71,72,85-89]. For instance, public posts can be utilized to measure prevalent happiness which can impact health through emotional contagion and the interconnectedness between mental health and physical health. Additionally, public posts about health behaviors may help us understand the prevalence of those behaviors as well as local area social norms. We demonstrate that tweets can provide a means to assess prevalent sentiment and food behaviors and physical activity, which can inform health interventions and policies to meet the needs of different neighborhoods. In particular, as this study suggests, neighborhoods with social and economic disadvantage, high urbanicity, and those with more fast food restaurants may exhibit lower happiness and fewer healthy behaviors.

## Appendix

Multimedia Appendix 1 Varying MALLET cut points for happy tweets and comparisons with manually generated labels.

Multimedia Appendix 2 National distribution of happy tweets, by zip code. Geotagged tweets were spatially joined to their 2010 zip code locations and sentiment scores were computed. This color coded map presents the proportion of happy tweets in each zip code area, with darker colors signifying higher proportions of happy tweets.

Multimedia Appendix 3 Proportion of happy tweets, by state.

## Abbreviations

API: application programming interface
BRFSS: Behavioral Risk Factor Surveillance System
MALLET: Machine Learning for Language Toolkit
Mturk: Mechanical Turk
NAICS: North American Industry Classification System
