# Supplementary material for: Building a National Neighborhood Dataset From Geotagged Twitter Data for Indicators of Happiness, Diet, and Physical Activity
Source: JMIR Public Health Surveill. 2016 Oct 17;2(2):e158. doi: 10.2196/publichealth.5869 (PMC5088343; doi:10.2196/publichealth.5869)
Supplement: Multimedia Appendix 1 [file publichealth_v2i2e158_app1.pdf]

eTable 1. Varying mallet cut points for happy tweets and comparisons with manually generated labels

	Accuracy	Prevalence of happy tweets
Mallet cut point ≥ 0.50	49%	68%
Mallet cut point ≥ 0.60	65%	48%
Mallet cut point ≥ 0.70	76%	31%
Mallet cut point ≥ 0.75	79%	25%
Mallet cut point ≥ 0.80	80%	19%
Mallet cut point ≥ 0.85	81%	14%

N=1200 control tweets; prevalence of happy tweets = 21%
Area under the ROC curve is approximately 0.7 for Mallet cut points between 0.6-0.85. Area under the ROC curve is 0.6 for a mallet cut point of 0.5
