# Supplementary figures and images for: Building a National Neighborhood Dataset From Geotagged Twitter Data for Indicators of Happiness, Diet, and Physical Activity
Source: JMIR Public Health Surveill. 2016 Oct 17;2(2):e158. doi: 10.2196/publichealth.5869 (PMC5088343; doi:10.2196/publichealth.5869)

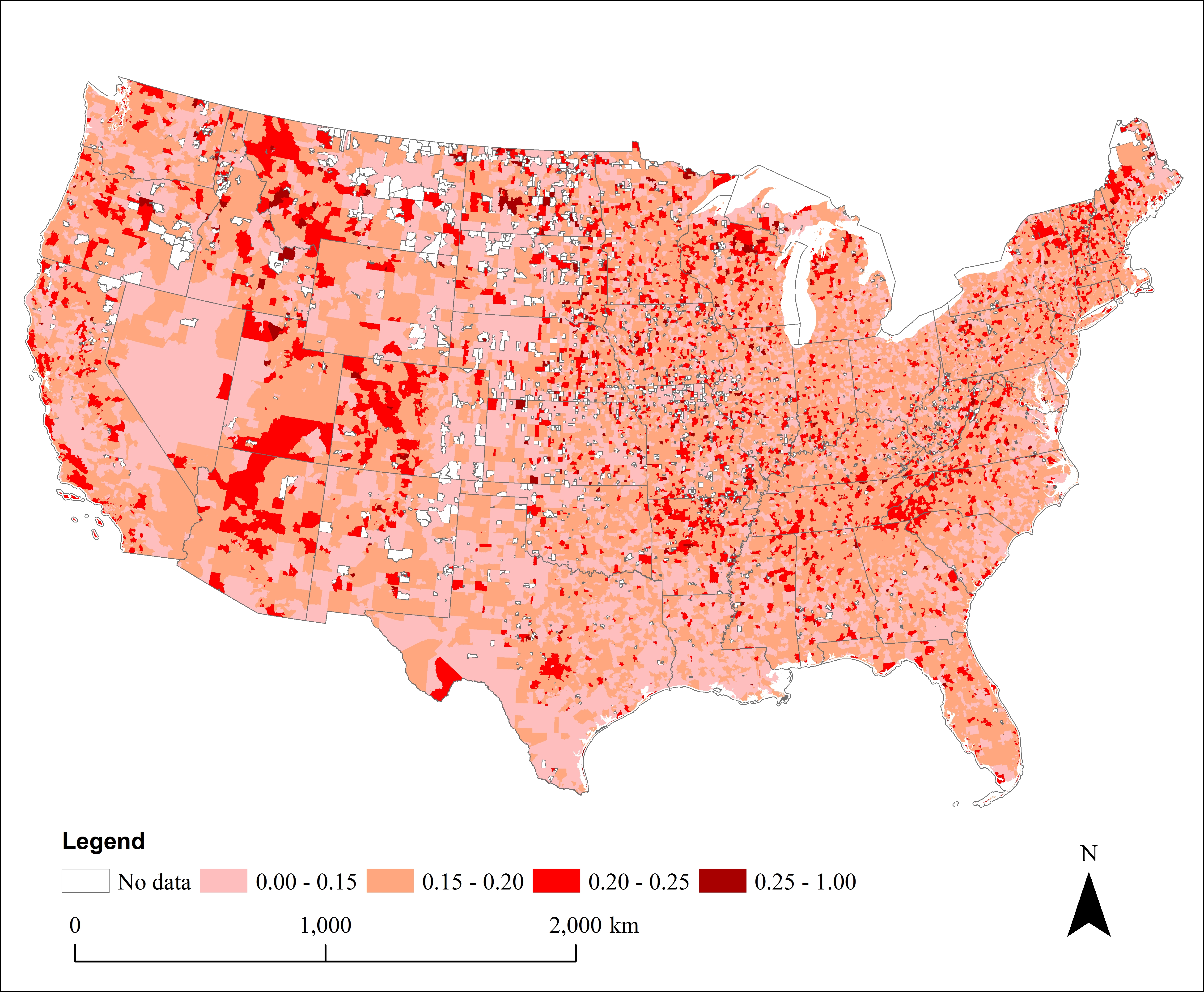

Supplement: Multimedia Appendix 2 [file publichealth_v2i2e158_app2.jpg]
