# Supplementary material for: Building a National Neighborhood Dataset From Geotagged Twitter Data for Indicators of Happiness, Diet, and Physical Activity
Source: JMIR Public Health Surveill. 2016 Oct 17;2(2):e158. doi: 10.2196/publichealth.5869 (PMC5088343; doi:10.2196/publichealth.5869)
Supplement: Multimedia Appendix 3 [file publichealth_v2i2e158_app3.pdf]

eTable 2. Proportion of happy tweets, by state

State	Proportion of tweets that are happy	Number of Tweets
Montana	24%	80018
Tennessee	24%	1385504
Utah	22%	422904
New Hampshire	22%	191452
Arkansas	22%	362849
Maine	22%	186208
Colorado	22%	903661
New York	22%	6329717
SouthDakota	21%	85074
Nevada	21%	1152492
Minnesota	21%	828800
California	21%	11169695
Vermont	21%	89930
Arizona	21%	1364823
Wyoming	21%	49840
Missouri	20%	979960
District of Columbia	20%	768767
Rhode Island	20%	241744
Florida	20%	5532007
North Carolina	20%	2145461
Wisconsin	20%	827090
Iowa	20%	469020
Idaho	20%	160570
Kentucky	20%	862995
South Carolina	20%	938297
Washington	20%	1397672
Massachusetts	19%	1562021
Illinois	19%	2663131
Michigan	19%	2208406
Nebraska	19%	306411
Georgia	19%	2531571
Indiana	19%	1268337
New Mexico	19%	259722
Alabama	19%	893865
Connecticut	19%	677048
Virginia	18%	1771281
Pennsylvania	18%	2730634
Kansas	18%	501238
Oklahoma	18%	730269

New jersey	18%	2175968
Mississippi	18%	461223
Ohio	17%	2756748
West Virginia	17%	308441
Delaware	17%	239194
Texas	17%	7313198
Maryland	17%	1586106
Oregon	16%	1029209
North Dakota	15%	129976
Louisiana	13%	1439345
